# Randomized control trial testing the effectiveness of implemented depression prevention in high-risk adolescents

**DOI:** 10.1186/s12916-020-01656-0

**Published:** 2020-07-24

**Authors:** Karlijn W. J. de Jonge-Heesen, Sanne P. A. Rasing, Ad A. Vermulst, Ron H. J. Scholte, Kim M. van Ettekoven, Rutger C. M. E. Engels, Daan H. M. Creemers

**Affiliations:** 1 GGZ Oost Brabant, P.O. Box 3, 5427 ZG Boekel, The Netherlands; 2 Erasmus School of Social and Behavioural Sciences, Erasmus University Rotterdam, P.O. Box 1738, 3000 DR Rotterdam, The Netherlands; 3 Child and Adolescent Studies, Utrecht University, P.O. Box 80125, 3508 TC Utrecht, The Netherlands; 4 Praktikon, P.O. Box 6909, 6503 GK Nijmegen, The Netherlands; 5 Behavioural Science Institute, Radboud University Nijmegen, P.O. Box 9104, 6500 HE Nijmegen, The Netherlands

**Keywords:** Adolescence, Prevention, Depression, School, Cognitive behavioral therapy

## Abstract

**Background:**

Adolescent depression is a global mental health concern. Identification and effective prevention in an early stage are necessary. The present randomized, controlled trial aimed to examine the effectiveness of Cognitive Behavioral Therapy (CBT)-based depression prevention in adolescents with elevated depressive symptoms. This prevention approach is implemented in school communities, which allows to examine effects under real-life circumstances.

**Methods:**

A total of 5222 adolescents were screened for elevated depressive symptoms in the second grade of secondary schools; 130 adolescents aged between 12 and 16 years old (*M* = 13.59; *SD* = 0.68; 63.8% girls) were randomly assigned to the experimental (OVK 2.0) or control condition (psycho-education). Self- and parent-reported depressive symptoms were assessed at pretest and post intervention, as well as 6- and 12-months follow-up. Clinical assessment of depression was assessed at pretest and 6-months follow-up.

**Results:**

Intent-to-treat analyses revealed that the decrease in adolescent-rated depressive symptoms was significantly larger in the intervention condition than in the control condition. There was no significant difference in decrease of parent-rated depressive symptoms between both conditions.

**Conclusions:**

Based on the findings, we recommend the implementation of screening and prevention in schools, according the basics of this study design. Since this is a new step forward, we discuss the clinical impact and challenges, as well possibilities for future research.

**Trial registration:**

The study is registered in the Dutch Trial Register for RCT’s (NTR5725). Date registered: 11 March 2016.

## Background

Depression is a common disorder that affects millions of people worldwide and is nowadays the leading cause of disease burden, according to the World Health Organization [1]. The onset of a depression often starts in adolescence with rates that increase substantially between 13 and 18 years of age [2]. In the Netherlands, the life time prevalence of a depressive disorder in this age group is 15.5%, and 8.8% experienced a depressive disorder in the past 12 months [3]. The early onset of depression, but also the presence of subclinical depression, affects the academic and interpersonal functioning and is associated with other psychopathologies, such as substance use, anxiety, and suicidality [4–7]. On the longer term, depression in adolescence is often linked to recurrent and chronic depressive episodes in adulthood [8, 9].

Consequently, prevention programs for adolescents receive growing attention. Several prevention programs worldwide were developed and examined on three levels: (1) universal prevention, which is aimed at all individuals; (2) selective prevention, which is aimed at individuals at risk for depression; and (3) indicated prevention, which is aimed at individuals with elevated depressive symptoms [10]. Overall, meta-analyses have established that depression prevention programs among adolescents are more effective in reducing depressive symptoms than usual care, waiting lists, or monitoring conditions with the largest effect sizes for selective and indicated prevention [10–13]. Although the effects are small to moderate and outcomes are heterogeneous, it seems important to continue with implementing and evaluating selective and indicated prevention programs for depression [11].

Studies so far have provided useful information about the effectiveness of specific elements that are covered in depression prevention programs. For example, we know that programs based on cognitive behavioral therapy and interpersonal treatment (IPT) have the largest effect sizes. Also, the duration of prevention programs can affect the magnitude of treatment effects, with shorter programs being more effective in reducing depressive symptoms [10, 13–15]. Moreover, factors concerning the delivery of prevention programs can improve the effects. For example, effect sizes for school-based programs are larger when delivered by a psychologist than school staff [13, 16, 17]. Despite this knowledge, there is limited evidence for the effectiveness of indicated depression prevention programs that are actually implemented in schools [11, 18].

Implementation of prevention programs seems to suffer from the large gap that exists between research and practice. Schools, for example, are often utilized for the examination of prevention programs, as this provides an easy way to reach adolescents and relieves most practical barriers, such as location, costs, and time [11, 19]. However, the implementation of prevention programs—in the context of research—is generally more ad hoc than the actual implementation of evidence-based programs in schools with the purpose of preventing symptoms of psychopathology. The process of transferring evidence into practice asks for an adequate infrastructure and engaging schools and individuals in the process of implementation takes a lot of effort [20]. Additionally, there are factors that may complicate implementation, such as poor financing, a lack of public awareness, and a non-supportive political atmosphere [21]. Therefore, rather than focusing on if, we should focus on how we can provide prevention programs in a sustainable manner.

To meet this ambition, we examined the effectiveness of depression prevention for high-risk adolescents when fully implemented in school communities. Prior to the study, an intensive collaboration was started between all the schools in a rural area in the south of the Netherlands, public health services, the caregivers within the schools, and mental healthcare services that were connected to the schools. We named this collaboration on depression prevention the STORM-project, which stands for Strong Teens and Resilient Minds. The following preventive interventions were included: (1) early screening on depressive symptoms and suicidal ideation; (2) detected suicidality, followed by clinical referral; and (3) an indicated prevention program of eight sessions based on CBT for adolescents with elevated depressive symptoms. All participating organizations had a part in the prevention process, with the public health service responsible for screening and referral; with the school’s licensed psychologists, together with caregivers from mental healthcare organizations, delivering the prevention program; and with the specialized mental healthcare institutes providing care for adolescents with high suicidal risk and supporting the process by sharing expertise and providing training.

Besides the benefits of collaboration on the communicating level, the continuity of care, and the expected reduction in overall mental healthcare costs, collaboration in the process of screening, identification, and prevention allows to identify and reduce depressive symptoms before they become severe. According to the clinical guidelines for the treatment of depression, collaboration is essential to ensure timely and effective access to help, and prevention programs should also benefit from collaboration [22–24]. Several recent meta-analyses made clear that collaborative care leads to better patient outcomes, reductions in health costs, and better patient and provider satisfaction [25, 26]. Although a number of studies proved the effect or effectiveness of CBT depression prevention programs by involving school staff or community providers into the delivery of the program [13, 27, 28], this is one of the first study that proved the effectiveness of indicated depression prevention with the process of screening and prevention implemented in the school community—based on a strong collaboration with schools and caregivers [10, 11, 15, 18].

### Present study

The purpose of the current study was to evaluate the effectiveness of a CBT depression prevention program—Op Volle Kracht 2.0 (OVK 2.0, which translates to At Full Force)—as implemented in school communities in the prevention of depression for adolescents with elevated depressive symptoms. A randomized controlled trial (RCT) was conducted, in which we screened adolescents on the presence of depressive symptoms and allocated participated adolescents with elevated depressive symptoms to the intervention and an active control condition, in which participants received psycho-education. The implementation of an infrastructure based on collaboration within school communities allowed us to evaluate OVK 2.0 under real world circumstances.

From a baseline to 12-month follow-up, as reported by adolescents and parents, we hypothesized that OVK 2.0 would lead to greater reduction in depressive symptoms, compared to psycho-education. Furthermore, we expected that adolescents who received OVK 2.0 would have a lower chance of depression onset after the intervention.

## Methods

The medical ethics committee CMO Region Arnhem-Nijmegen of the Netherlands approved this study (NL55328.091.15). The study is registered in the Dutch Trial Register for RCT’s (NTR5725). The study design will be reported in accordance with the CONSORT 2010 statement for reporting parallel group randomized trials [29].

### Procedure

A total of 5222 adolescents in the second year of 13 secondary schools, from vocational training up to pre-university level, were screened on depressive symptoms during two consecutive school years (October–March 2016/2017 and October–March 2017/2018) with the Children’s Depression Inventory 2 (CDI-2) [30, 31]. The screening was performed by the public health service (in Dutch: GGD) and was part of a larger routine health survey.

**Fig. 1:**
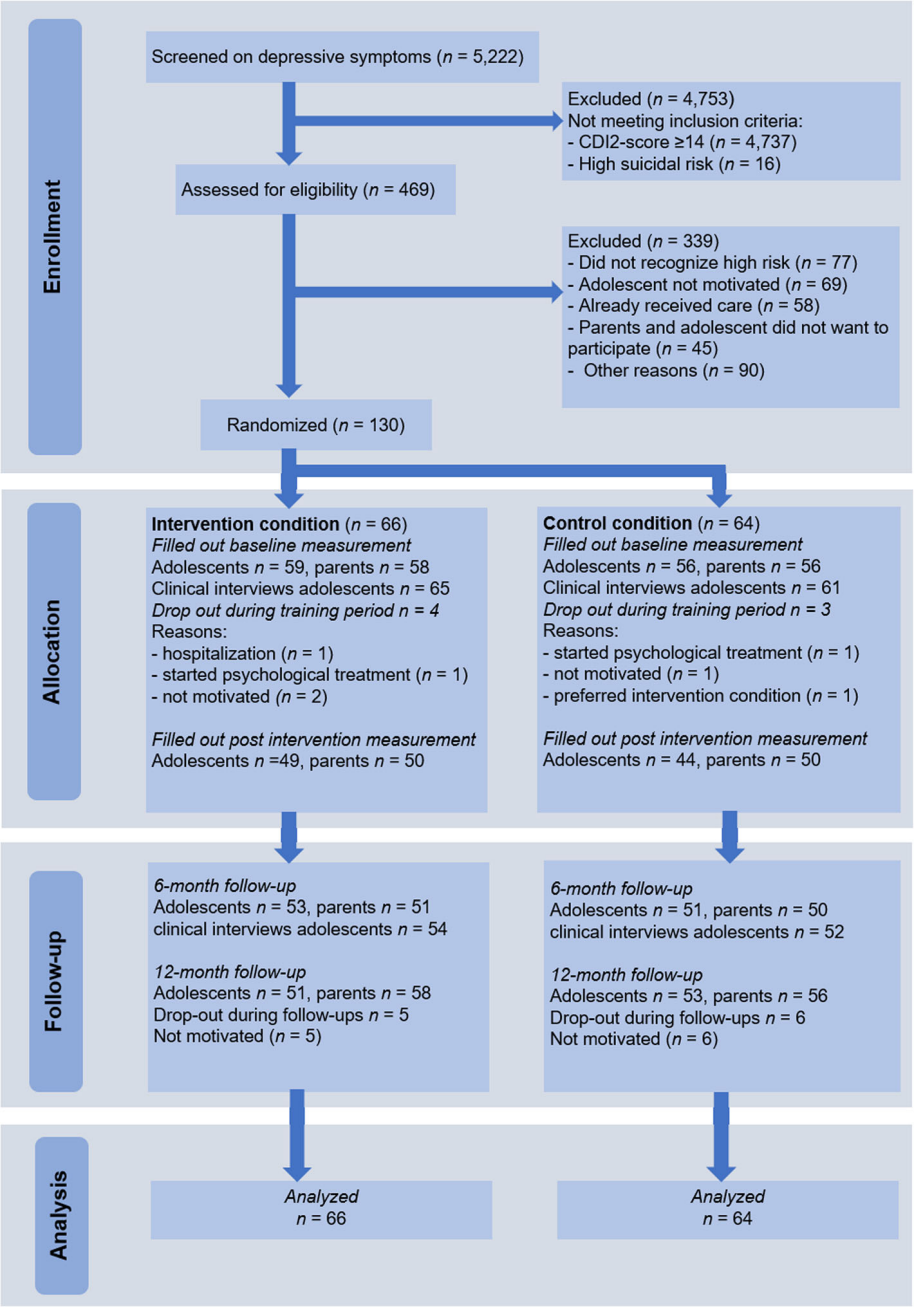
Flow diagram of participants

Inclusion criteria were elevated depressive symptoms according to the screening (score ≥ 14 CDI-2) [30], ages between 11 and 15 years old, and sufficient knowledge of the Dutch language to participate. Exclusion criteria were the absence of parental permission, already undergoing CBT for mood problems, or the presence of high suicidality. Sixteen adolescents (0.3%) presented high suicidal ideation, and they were referred to mental healthcare organizations. All 457 (8.7%) adolescents who scored above the cutoff of 14, along with their parents, received information regarding the study. Next, adolescents and parents were contacted by the research team. After receiving informed assent from adolescents and parents, participants were randomly allocated to the experimental or control condition, stratified on school level. The randomization was carried out within schools by an independent researcher using a computer-generated randomization procedure. Adolescents and parents completed online surveys at the baseline (T1), after the intervention (T2), at 6-month follow-up (T3), and at the 12-month follow-up (T4). In addition, adolescents received a semi-structured clinical interview at the baseline and 6-month follow-up to determine the presence of clinical depression. Participants were informed of group allocation before the baseline measurement (T1). Adolescents received gift vouchers as a reward for filling in the questionnaires. More information about the procedure and participant flow is provided in Fig. 1.

When participants during the study appeared to be at high risk for suicidality (as appeared from the questionnaires) or at risk for a severe clinical depression (as measured by the interview at the 6-month follow-up), they were seen by a professional of the public health service, parents were informed, and eventually, information about referrals were provided.

### Sample size and power

The intended main analysis strategy, as described in the study protocol [32], was a repeated measures ANOVA. To find a significant difference in depressive symptoms between conditions, a sample size of 78 was needed (type I error probability of 0.05, power = 0.80, and medium effect size of *f* = 0.25). Including the effect of clustering data (group effect because the intervention was given in small groups), and an ICC of 0.07 in a comparative study [33]), the variance inflation factor became 1.49. The sample size had to be raised to 117 participants. Including the possible drop out of participants, the intended sample size was 160. The choice for an effect size of *f* = 0.25 was based on comparable studies [11, 33]. Regarding the analysis method, latent growth curve modeling (LGCM) [34] was planned as an additional strategy but was chosen as the main strategy above repeated measures ANOVA, as this is a more flexible approach and directed to individual change over time. Given that repeated measures ANOVA is comparable with LGCM, we assumed that the power of the two techniques is about the same.

### Participants

Out of the 5222 adolescents, 469 (62.7% girls) had elevated depressive symptoms and were approached for participation in the study. Finally, 130 adolescents (63.8% girls) with elevated depressive symptoms participated. The participants were aged between 11 and 15 years (*M* = 13.59; *SD* = 0.68). Educational levels were as follows: vocational training (45.4%), vocational or high school training (5.4%), high school training (17.7%), high school or pre-university training (2.3%), and pre-university training (19.2%). Most participants were of Dutch origin (85.4%). Of the participants, 59.2% lived with their biological parents, and the other participants had divorced parents or a different family situation and lived, for example, with their foster parents.

### Interventions

#### OVK 2.0

The Penn Resiliency Program (PRP) (30), which proved to be effective as a school-based universal prevention in the United States (US), was adapted to the Dutch culture and called OVK [35, 36]. The goal of this CBT-based prevention program is to teach adolescents to identify thoughts and emotions, as well as how activating events, thoughts, emotions and behaviors are related. In contrast to PRP, the OVK program did not prevent or decrease depressive symptoms on an universal and selected level [37, 38], but it did prove to be effective among adolescent girls when used as indicated prevention in a shortened protocol (i.e., 8 lessons instead of the original 16) [33].

OVK 2.0 is a modified and more up-to-date version of the original OVK program. Equal to the study of Wijnhoven and colleagues [33], it consists of eight weekly, 1-hour lessons in groups of three to eight adolescents. These eight lessons are based on CBT techniques. Homework in OVK 2.0 includes mood monitoring and energizing assignments that are based on positive psychology. Moreover, multi-media sources are included. Before the start of the program, there was an individual intake with the adolescent and the trainers and an information meeting for the adolescents and their parents. Three months after the program, the trainers organized a booster session for the adolescents and their parents. More details about the content of the program are described in a protocol paper [32].

The program was delivered by school psychologists who were also staff members at school, together with a co-trainer of the collaborated mental healthcare organizations. They all received a 3-day training program, covering training in CBT skills, theoretical principles, and the use of the prevention protocol. We measured treatment fidelity through a self-report questionnaire, assessing which exercises were actually given and that trainers had to fill in after each lesson. The treatment fidelity was 84.7% (range from 74.6 to 94.7%).

#### Psycho-education

The participants in the psycho-education condition received a brochure with information about depressive symptoms. Also, participants received two e-mails with useful tips to boost their mood and decrease depressive symptoms. For example, they were encouraged to do more physical exercises and to find a sport they might like.

### Measures

*Depressive symptoms* were measured with the CDI-2 [30, 31]. The CDI-2 is a self-report questionnaire comprising 28 items, each consisting of three statements rated in severity from 0 to 2 (e.g., “I don’t feel alone” = 0, “I often feel alone” = 1, “I always feel alone” = 2). The CDI-2 was used for screening purposes, in accordance with the Dutch clinical guidelines for depression among youth [24]. The CDI-2 has good psychometric qualities [39]. In this study, Cronbach’s alpha ranged between 0.78 and 0.89.

*Depressive symptoms according to parents* were measured with the Dutch translation of the parent version of the CDI-2 [30, 31]. This questionnaire comprised 17 items that are measured on a 4-point scale from 0 (not at all) to 3 (almost always), and parents had to rate the extent to which the items were in accordance with their child’s thoughts, feelings, and behaviors (e.g., “My child seems lonely”). The psychometric qualities of the parent version of the CDI-2 were good [39]. In this study, Cronbach’s alpha ranged between 0.78 and 0.85.

*The presence of a clinical depression* was measured by the Dutch version of the Anxiety Disorder Interview Schedule for Children (ADIS-C) [40, 41] during a clinical interview. This is a semi-structured diagnostic interview of the symptomatology, course, and severity of anxiety, mood disorders, and externalizing disorders in 7 to 17-year-old children, according to the DSM IV diagnostic criteria. In the study, we included a separate section relating to mood disorders, which includes the diagnostic criteria for dysthymic disorder and major depressive disorder. The interview took about 10 to 20 min, and it was administered by a qualified psychologist or by a trained master student under the supervision of a qualified psychologist. Participants had to respond “yes,” “no,” or “different” to standardized questions. The purpose of this interview was to investigate whether children met the criteria for a dysthymic disorder or major depression disorder. Interrater reliability and test-retest reliability of the ADIS-C were found to be good [42].

*Suicidality* (i.e., the presence of suicidal ideation) was measured with item 8 of CDI-2 on a three point scale (0 = “I don’t think about ending my life,” 1 = “I think about ending my life, but I would never do it,” and 2 = “I want to end my life”). When adolescents reported a score of 2 on this item, during the screening or follow-ups, they were approached for an assessment by professionals of the public health service. These professionals were trained in the assessment of suicide risk by the project staff. In this assessment, the presence and severity of suicidality was checked, parents were informed, and clinical referrals were provided when necessary. During the screening, 54 adolescents scored 2 on the CDI-2 and were subsequently interviewed. Of these adolescents, 31 (57.4%) made a mistake, were joking, or misinterpreted this item; 7 (13.0%) adolescents were at low risk for suicidality and were approached for participation; 3 (5.5%) adolescents were at high risk for suicidality and needed to be referred to mental healthcare; 10 (18.5%) adolescents were at high risk for suicidality and received already mental healthcare and, in some cases, upscaling of care was necessary and this was discussed with the involved care provider; and 3 (5.5%) adolescents were at such high risk for suicidality that immediate help was necessary. When adolescents reported suicidality on follow-up measurements, they were contacted by the research team and invited for an assessment by the public health service. These participants were not excluded from the study.

*Demographical variables* were gender, educational level (i.e., vocational training or higher education), ethnicity (i.e., whether born in the Netherlands), and family situation (i.e., living with biological parents or different living situation).

### Strategy of analyses

#### Attrition

We conducted logistic regression analyses to analyze attrition at screening (T0; *n* = 465) trough baseline (T1; *n* = 130). Enrollment was used as the dependent variable, and depressive symptoms levels at screening and gender were used as predictors. The results indicated no differences for gender (*OR* = 0.88; *p* = 0.546) or depressive symptoms level (*OR* = 0.98; *p* = 0.455). Attrition was also analyzed for adolescents who were labeled as drop-outs because they did not fill in the questionnaires at T2, T3, and T4 or withdrew early in the study. No significant effects were found for the following: condition (*OR* = 0.67; *p* = 0.671), gender (*OR* = 0.43; *p* = 0.378), school level (*OR* = 4.50; *p* = 0.193), and ethnicity (*OR* = 1.68; *p* = 0.682).

During the study, we became aware of suicide attempts by two participants. One was reported by the mother in the parent questionnaire, and the other was informed by one of the collaboration partners. Both were girls, one in the experimental condition and one in the control condition. We reported these suicide attempts as serious adverse events.

### Analyses

First, descriptive statistics and *z*-tests were used to describe and to analyze differences in depressive symptoms at all time-points for adolescents and their parents, with help of the statistical package, Mplus [34].

The data were analyzed according to the intent-to-treat principle. To examine change in depressive symptoms over time, we used LGCM with Mplus [34]. Missing data were handled by using the full information maximum likelihood estimator [43, 44]. Participants with missing data on all four time points were automatically excluded from the analyses (5 adolescents, 11 parents). A prerequisite to use this estimator is that missing values of depressive symptoms are missing at random. Little’s MCAR test showed that completely missing at random and, therefore, also missing at random is supported (*χ*^2^ (41) = 47.46; *p* = 0.226).

To control for possible non-independence of the data because of nesting participants within schools, the procedure COMPLEX with the robust maximum likelihood estimator (MLR) was used. With this procedure, we got unbiased standard errors of the parameter estimates. Model fit indices were chi-square (*df*), the root mean square of approximation (RMSEA; values < 0.08 means acceptable fit) [45], and the Comparative Fit Index (CFI; values > 0.90 means acceptable fit) [46]. Although these global fit indices are less suited for small sample sizes (*N* < 200) [47, 48], Coffman and Millsap found that despite a poor global fit [49], a linear growth model may provide a good approximation of the actual growth curve. Therefore, in the first step, we examined the adequacy of a linear model in comparison to a quadratic model for adolescent ratings of depressive symptoms. A linear model for both groups showed a fit of *χ*^2^ (10) = 24.13, *p* = 0.007, RMSEA = 0.151, CFI = 0.907. The fit was acceptable according to the CFI-value and less acceptable according to the RMSEA value. A quadratic model for both groups showed a fit of *χ*^2^ (2) = 0.46, *p* = 0.793, RMSEA = 0.000, CFI = 1.000. The fit of this model was excellent, but the question arose whether for this small sample of *N* = 130, a quadratic model was overfitting the data [50]. Therefore, we examined how well the estimated depressive symptoms scores at T1, T2, T3, and T4 (according to a linear or a quadratic regression model), correlated with the original scores on depressive symptoms. For the linear model, the correlations were 0.86, 0.86, 0.85, and 0.97, and for the quadratic model, the correlations were 0.98, 0.78, 0.98, and − 0.01. At T4, the correlation between the original scores and the estimated scores was zero, and this was an indication that a quadratic model is overfitting the data. For this reason, a linear model was chosen for adolescent-rated depressive symptoms as most adequate for our purposes, with intercept (*i*; initial estimated level of depressive symptoms) and slope (*s*; estimated degree of change of depressive symptoms over time) as latent growth parameters.

For parent-rated depressive symptoms, the fit of the linear model was *χ*^2^ (10) = 24.60, *p* = 0.006, RMSEA = 0.157, CFI = 0.913. The fit was acceptable according to the CFI-value and less acceptable according to the RMSEA value. The fit for the quadratic model was *χ*^2^ (10) = 0.42, *p* = 0.810, RMSEA = 0.000, CFI = 1.000. Despite this excellent fit, we found that for the linear model, the correlations between original scores and estimated scores were 0.90, 0.96, 0.86, and 0.97, and for the quadratic model, the correlations were .98, .86, .98, and −.09, indicating that a quadratic model was overfitting the data. This supported the choice for a linear growth model. To test differences in *i* and *s* between the experimental and control condition, we used the *χ*^2^ difference test and compared the *χ*^2^ value of the unconstrained growth model with the *χ*^2^ value of the growth model, where the *i* was constrained to be equal in both conditions. A significant increase of the *χ*^2^ value from baseline to *i*-constrained model indicates a significant difference of the mean intercept. In the same way, the *χ*^2^ value of the *i*-constrained model was compared with the *χ*^2^ value of the model, where both *i* and *s* were constrained to be equal. A significant difference in *χ*^2^ value between both models indicates a significant difference in mean slope. As a result of using the MLR estimator, the *χ*^2^ values of the models were divided by a scaling correction to get a better approximate of the *χ*^2^ values. However, the difference between these two corrected *χ*^2^ values is not *χ*^2^ distributed. Therefore, the *χ*^2^ values of the models were first rescaled to uncorrected *χ*^2^ values before calculating the difference between the two *χ*^2^ values [51]. Effect sizes for treatment efficacy were calculated by the difference between the estimated means of the intervention and control condition at the end of the study (determined from the coefficient for the slope differences and length of study) divided by the baseline pooled standard deviation [52].

### Additional analyses

We have performed additional analyses to examine individual change over time of depressive symptoms. We calculated the Reliable Change Index (RCI) for each participant by dividing the pretest (T1) to follow-up (T4) score difference by the standard error of this difference. Adolescents with RCI scores above 1.96 were classified as significantly improved, adolescents with RCI scores between − 1.96 and 1.96 were classified as unchanged, and adolescents with RCI scores below − 1.96 were classified as significantly worsened [53]. We used Fisher’s exact test, followed by post hoc tests including Bonferroni correction (to correct for capitalization on chance), to test whether and how the classification of the intervention group differed from those of the control group. Furthermore, remission status of the participants that meet the criteria of a depressive disorder at baseline were calculated at the 6-month follow-ups, and the binary logistic regression analyses (with remission status as dependent variable and condition as predictor) were used to compare remission status between the experimental and control condition.

## Results

### Descriptives

In total, 5222 adolescents were screened on depressive symptoms. Of the 469 adolescents that emerged from the screening, 130 participated in the study and were included in the analyses. Participation rates were good (T1 = 88.5%; T2 = 71.5%; T3 = 80.0%; T4 = 80.0%), with a lower percentage at T2, probably due to the start of the summer holiday. Table 1 shows descriptive statistics and test results of a comparison between experimental and control condition for depressive symptoms (adolescent and parent rated) at all time points. No significant differences were found in adolescent-rated depressive symptoms between both conditions at T0, T1, T2, and T3. At T4, adolescents in the experimental condition reported significantly fewer depressive symptoms than adolescents in the control condition. Parent-rated depressive symptoms differed significantly between both conditions at all time points, with higher means in the experimental condition.

**Table 1:** Means, standard deviations, and *z* values for differences on adolescent-rated (CDI-2:C) and parent-rated depressive symptoms (CDI-2:P) between the intervention and control condition

	Intervention condition (*N* = 66)		Control condition (*N* = 64)			
**Adolescent-rated**	*M*	SD	*M*	SD	*z* value	*p*
CDI-2:C T0	18.66	4.21	18.42	4.54	.41	.679
CDI-2:C T1	16.18	4.92	15.68	7.08	.45	.655
CDI-2:C T2	13.32	7.07	14.71	9.06	− .80	.423
CDI-2:C T3	12.10	6.85	13.72	8.72	− .98	.326
CDI-2:C T4	10.78	7.05	13.32	7.50	− 1.98	.048
**Parent-rated**	(*N* = 60)		(*N* = 59)			
CDI-2:P T1	20.14	5.54	16.78	5.86	2.57	.010
CDI-2:P T2	18.66	6.00	15.09	6.36	2.96	.003
CDI-2:P T3	17.42	6.27	14.41	6.21	2.94	.003
CDI-2:P T4	16.59	6.34	14.07	6.53	2.10	.035

### Latent growth curve modeling

**Fig. 2:**
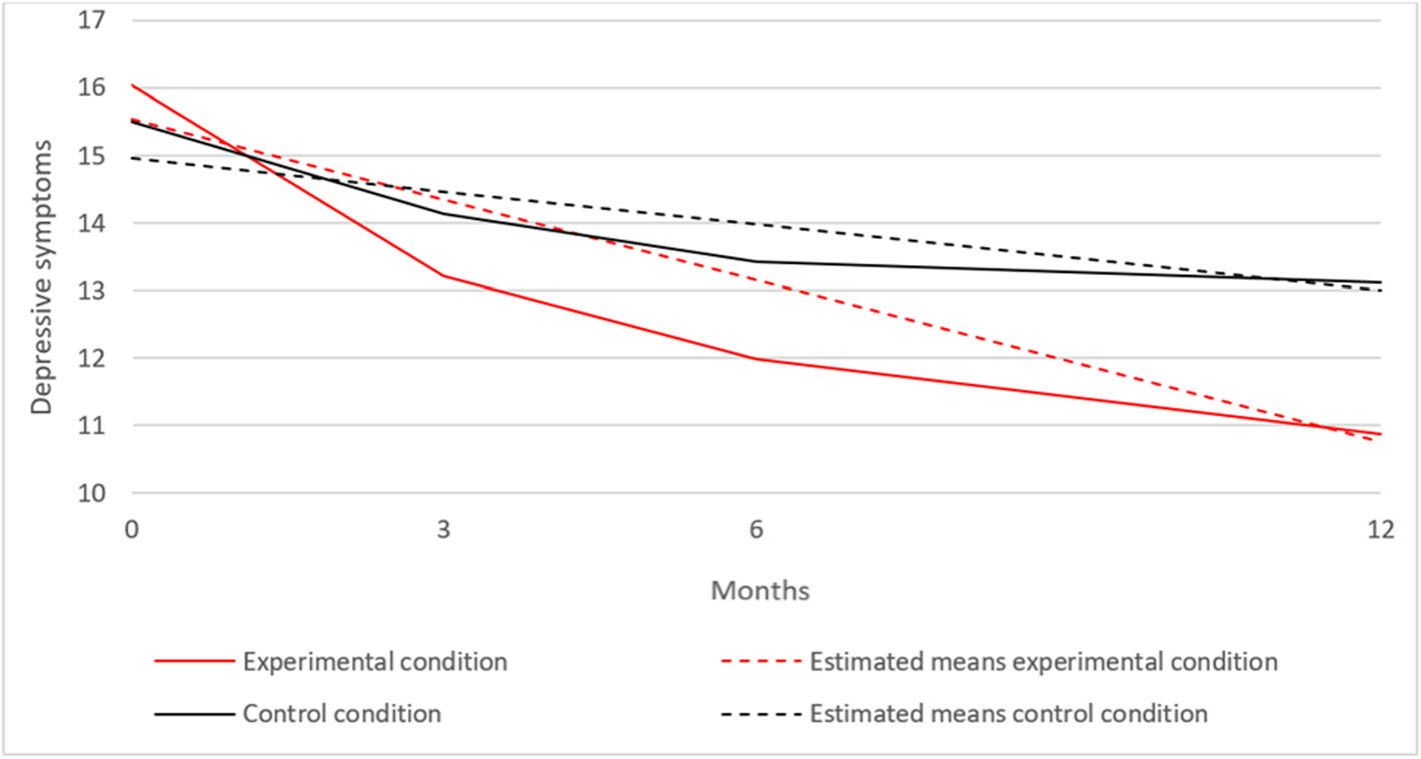
Mean scores of adolescent-reported depressive symptoms over time in the experimental and control condition

In the analysis section, it is concluded that a linear growth model for depressive symptoms is most appropriate for adolescent and parent ratings. Therefore, we examined both linear growth models in which time was coded in months (0, 3, 6, and 12 months). For adolescent ratings, the estimated growth parameters in this model were: *i* = 15.54 and *s* = − 0.40 (*p* = < 0.001) in the intervention condition and *i* = 14.95 and *s* = − 0.16 (*p* = < 0.001) in the control condition. Figure 2 shows this model. The second step was to test our hypothesis that the intervention condition would show a greater decrease in depressive symptoms than the control condition. First, we tested whether the intercept was different between both conditions. The *χ*^2^ difference test was not significant, with Δ*χ*^2^(1) = 0.07 and *p* = 0.791, indicating that the mean starting level of depressive symptoms was not significantly different between both conditions. Next, we tested whether the slope was different between both conditions. The *χ*^2^ difference test showed a significant effect, with Δ*χ*^2^(1) = 4.35 and *p* = 0.037, indicating that the decrease in depressive symptoms was larger in the intervention condition compared to the control condition. The effect is 0.47, indicating an almost medium effect size.

**Fig. 3:**
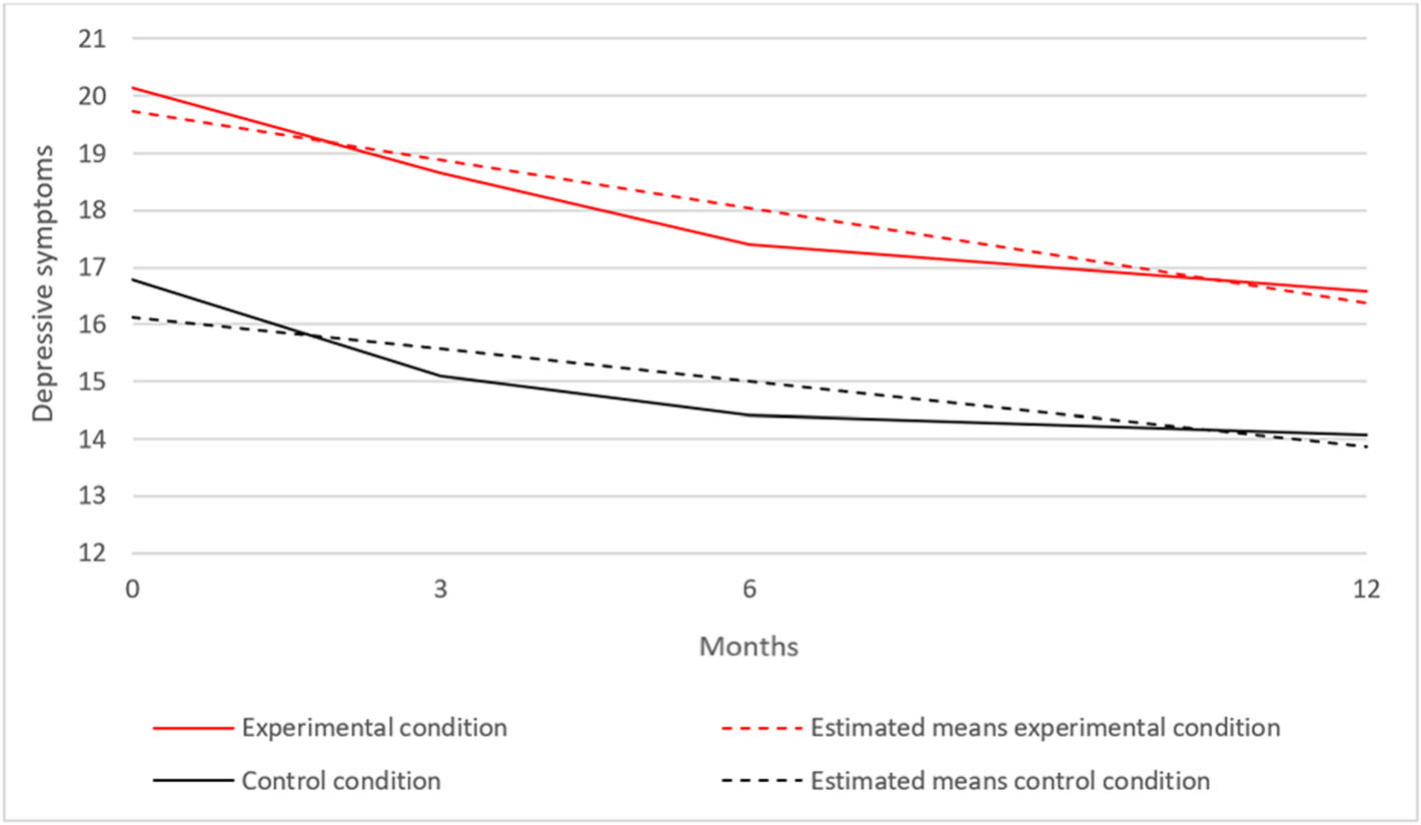
Mean scores of parent-rated depressive symptoms over time in the experimental and control condition

For parent ratings, the parameters were *i* = 19.72 and *s* = − 0.28 (*p* = < 0.001) in the intervention condition, and *i* = 16.14 and *s* = − 0.19 (*p* = 0.018) in the control condition. Figure 3 shows this model. The intercept appeared to be significant between both conditions, and the *χ*^2^ difference test showed a significant effect for the intercept with Δ*χ*^2^(1) = 10.76 and *p* = 0.001. The mean starting level of depressive symptoms in the intervention condition is significantly higher than in the control condition. There was no significant difference in decrease of parent-rated depressive symptoms between both conditions (Δ*χ*^2^(1) = 1.75; *p* = 0.186). The effect size is 0.19, indicating a very small effect size.

### Individual change over time

Between the baseline and 12-month follow-up, 38.3% of the adolescents in the intervention condition improved, compared to 12.5% in the control condition. The percentage of adolescents that worsened was 2.1% in the experimental condition, compared to 8.3% in the control condition. The percentage of adolescents that remained unchanged was 59.6% in the intervention condition and 79.2% in the control condition. Fisher’s exact test showed a *p* value of 0.007, indicating that the control condition differed from the intervention condition. Post hoc test including Bonferroni correction showed that in the intervention condition, the percentage of participants that did not change was significantly lower, and the percentage of participants that improved was significantly higher than in the control condition.

### Remission status

Furthermore, we calculated the remission status of participants that met the criteria of a depressive disorder in both conditions. Only participants for whom pretest and the 6-month follow-up measure of the ADIS-C were available were included in the analysis. A total of 105 participants met this criterion. At pre-test, 7 participants (10.8%) in the intervention condition and 12 participants (19.7%) in the control condition fulfilled the diagnosis of a depressive disorder. At the 6-month follow-up, 3 participants (5.5%) in the intervention condition and 4 (7.7%) in the control condition fulfilled the diagnosis of a depressive disorder. Binary logistic regression analyses revealed that remission status did not differ between conditions (*OR* = 0.83, *p* = 0.906).

## Discussion

The aim of the present study was to test the effectiveness of OVK 2.0 in the prevention of depression for adolescents with elevated depressive symptoms when implemented in school communities. Findings from LGCM show that the decrease in self-reported depressive symptoms was significant in both conditions, from the baseline to 12 months after the interventions, and that the decrease in depressive symptoms was significantly larger in the experimental condition with 42.6% of the adolescents who improved, than in the control condition with 16.2% of the adolescents who improved. This indicates that a CBT prevention program is effective in reducing elevated depressive symptoms in adolescents when implemented in school communities.

Based on the findings, we recommend the implementation of screening and prevention in schools according the basics of this study design, which is screening on depressive symptoms and suicidal ideation and providing indicated prevention. Although psycho-education could be a low-cost and easier way for offering prevention, the findings indicate that CBT-based prevention might ensure more sustainable outcomes. Although this study did not examine the advantages or benefits from the integrated care approach that formed the base of this study, we experienced that there are a number of foreseen as well unforeseen profits. In addition to the improved collaboration between schools and care providers, we were able to identify and refer adolescents high at risk for suicidality. Important as well, we experienced that, in both conditions, the confidence of schools and care professionals regarding this topic increased over the course of the study and that adolescents felt more comfortable to display their worries or feelings within the school setting. Moreover, schools became activated in their role in prevention. Where in the first year of this project schools and care givers were hesitant, in the second year, they took initiative by planning for the next years and suggesting improvements. Also, schools initiated collaboration on other mental healthcare themes, such as self-harm. This way, such collaboration might form a stable infrastructure in which new developments can be adapted to improve prevention.

The positive findings regarding the effects of OVK 2.0 are promising in many ways. First, the findings are in line with Wijnhoven and colleagues [33], who found similar effects for OVK 2.0 in a sample of high-risk adolescent girls. This is important because we are often unaware of the relative effectiveness or circumstances that modify the effectiveness; therefore, it is likely that effects might not sustain when transferring into practice. This study showed that OVK 2.0, when part of implemented depression prevention, was also effective in reducing depressive symptoms in a mixed sample with boys and girls and when provided by different trainers. Second, although meta-analyses show that indicated prevention seem to be effective in the short term only [10, 15], this study showed that the effect remained at the 12-month follow-up. Lastly, to our knowledge, this is the first study that examined indicated depression prevention in adolescents when implemented in the school community; therefore, the findings give hope for future implementation.

Despite the significant decrease of self-reported depressive symptoms in the OVK 2.0 condition, there was no significant difference in the decrease of parent-rated depressive symptoms. Also, there was no significant difference between conditions in future incidence of depressive episodes. Although the study is underpowered to detect differences in depression onset, it is encouraging that, given the sharp increase in depression rates during adolescence, depressive symptoms actually decreased during the study.

Furthermore, a discrepancy between parents and adolescents in the report of depressive symptoms is frequently observed, with parents over-reporting in a general population and underreporting in a clinical population [30, 54]. Parents seem to be more aware of symptoms when they become severe, possibly because symptoms then become visible. This could also explain the higher starting level of parent-reported depressive symptoms in the intervention condition, as compared to the control condition. By allocation to the intervention condition, parents might be more aware of symptoms and a possible change due to the active character of the intervention than in the relatively passive control condition. Also, nonresponses and the different reasons for this nonresponse might have caused the observed difference. For example, high parent-rated scores might be missing due to disappointment of parents that their child did not made it to the intervention condition. Nevertheless, future research should continue to include both informants as a study of Cohen and colleagues [55] showed that adolescent-reported symptoms are the best predictors for concurrent depressive episodes; however, to predict future episodes, both adolescent and parent reports were necessary. Parents seem to be better in identifying behavioral signs that are important precursors of a depression.

### Strengths and limitations

This study has some important strengths. First, we implemented the evaluated preventive interventions in school communities, which allowed us to examine the effects of OVK 2.0 under real-life circumstances. Second, we used adolescent-rated and parent-rated depressive symptoms. Moreover, we used a clinical interview to determine the effects of depression prevention on actual prevention of diagnoses of depression. Third, the study design allowed us to examine the effectiveness of OVK 2.0 beyond an active control condition. Finally, the follow-up measurements allowed us to examine the long-term effectiveness of the intervention.

Despite the robust RCT design, some limitations must be noted. Randomization was carried out at the school level, which limits the random allocation of participants. Also, in the control condition, we did not measure if adolescents actually read the psycho-education and whether they found this information helpful, which limits the fidelity of this intervention. Furthermore, only 27% of the adolescents that were approached to participate in this study agreed to participate. This raises questions about a bias in selection of the participant group. For example, we noticed that parents in this age group had an important voice in whether to participate. A relatively large group of adolescents were initially not very motivated for participation, and it was up to their parent(s) if participation was refused or reconsidered. When depressive symptoms were not recognized in everyday life, it was more likely that they refused to participate (see also Fig. 1). Therefore, it might be that participated adolescents had more concerned parents or parents that endorsed the importance of prevention. This parent’s concern might also be displayed by the higher parent-rated depressive symptoms, as compared to adolescent-rated symptoms. Although the sample size was too small to rule out the effect of selectivity, future implementation studies should search for strategies to maximize enrollment.

### Clinical implications

Integrating depression prevention in (school) communities is a new step forward that brings new questions and challenges. As discussed in the limitations, motivating adolescents and parents for participation is one of those challenges. In order to improve enrollment, it is crucial to examine how we can improve the acceptability of prevention programs which might lower the threshold to join these programs. We experienced that the most important reasons to decline participation were the lack of motivation and the lack of understanding of utility and necessity of adolescents or parents. It is important to examine the best approach to increase the motivation and the sense of urgency, for example, by decreasing the stigma of depression among adolescents. Universal mental health programs could create more awareness of mental health and promote help-seeking behavior in adolescents [56], especially when this is integrated into the school curriculum.

In addition to the adolescents, it also seems important to create awareness in the adolescent’s environment, for example, by introducing parent information sessions in schools regarding this theme. Also, teachers and mentors, who are often in contact with both adolescents and parents, should be able to recognize depressive symptoms and support in the participation in prevention programs or the process of help-seeking. The gatekeeper training, for example, can be used for this purpose. Although the main goal of this training is to learn how to respond to suicidality, it proved to be helpful in referring adolescents to appropriate mental health services, especially when given in school-based settings [57, 58]. Moreover, the training improves knowledge of suicidality and confidence to conduct a dialog on suicidal thoughts [57]. Given the strong association between the presence of suicidality and depressive symptoms, as well as the screening on suicidality as a prevention strategy, the gatekeeper training might be a sufficient tool to engage teachers and mentors in depression and suicide prevention.

### Future research

This study showed an overall decrease in depressive symptoms for adolescents receiving the intervention. However, we have also shown that not all individuals benefited from the prevention program. The next step in research should be to gain more insight in for whom this strategy is effective, often mentioned as precision prevention. Earlier research, specifically longitudinal studies, identified four classes of adolescents, with each class characterized by a pattern of change of depressive symptoms: (1) low stable or no symptoms, (2) intermediate symptoms that decrease over time, (3) intermediate symptoms that increase over time, and (4) chronic or persistent high level of symptoms [59, 60]. The knowledge of these development patterns of depressive symptoms should be used to explore the effect of the intervention and to study if we could predict whether individuals respond to prevention programs. Another suggestion is to study whether certain risk factors or risk patterns predict the responsiveness of individuals to a prevention program. We expect that the chronic or persistent group includes adolescents at risk who are almost resistant for prevention programs due to a combination of risk factors (e.g., parental psychopathology, obesity, or traumatic childhood experiences). Future research should focus on the identification of these risk patterns, as this can be used to advance the screening and prevention process, for example, by combining parent and adolescent depression treatment or timely upscaling of mental healthcare. These suggestions should result in more insights into for whom the intervention will be effective or not and is necessary to take precision prevention to the next level.

## Conclusions

In conclusion, this study showed that a CBT program, as indicated depression prevention, is effective in reducing depressive symptoms when implemented in school communities. Given the high rates of adolescents that suffer from depressive feelings worldwide, implementation of indicated prevention programs, screenings, and subsequent referrals of suicidality are priorities. Schools, care givers, politicians, communities, and researchers should cooperate in the sustainable implementation of depression prevention. Bridging the gap between science and practice is challenging; however, with the opportunity to improve prevention work, the challenge is worth pursuing.

## Data Availability

The data for the current study is not publicly available due to them containing information that could compromise research participant privacy, but they are available from the corresponding author upon reasonable request.

## Abbreviations

ADIS-C: Anxiety Disorder Interview Schedule for Child
CBT: Cognitive behavioral therapy
CDI-2 C: Children’s Depression Inventory 2 child rated
CDI-2 P: Children’s Depression Inventory parent rated
CDI-2: Children’s Depression Inventory
CFI: Comparative Fit Index
IPT: Interpersonal treatment
LGCM: Latent growth curve modeling
MLR: maximun likelihood estimator
OVK: Op Volle Kracht
PRP: Penn Resiliency Program
RCI: Reliable Change Index
RCT: Randomized controlled trial
RMSEA: Root mean square of approximation
WHO: World Health Organization
